# The use of parasites as bioindicators of pesticide exposure

**DOI:** 10.2478/v10102-009-0015-1

**Published:** 2009-09-28

**Authors:** Rastislav Sabo, Lucia Sabová, Jaroslav Legáth

**Affiliations:** Department of Pharmacy, Pharmacology and Toxicology, University of Veterinary Medicine, Komenského 73, 041 81 Košice, Slovak Republic

**Keywords:** pesticides, parasites, exposure, bioindicators

## Abstract

Organisms used in risk assessment of pesticides must be the most sensitive ones to pesticides exposure. The aim of this study was to observe the effect of two commercial pesticide products (containing glyphosate and tolylfluanid) to larval stages of parasites *Cooperia curticei, Ostertagia circumcincta, Haemonchus contortus* and *Trichostrongylus axei*. There were two concentrations tested for each product vs. control group. Larvae (500 individuals/Petri dish) were incubated at 27 °C and observed daily for 42 days.

We found out that T. axei larvae are the most resistant ones to tolylfluanid exposure – there was no statistical significance in any concentration tested after 42 days of tolylfluanid exposure. 100% of dead larvae were found on 33^rd^ day of experiment at higher concentration, resp. on 37^th^ day at lower tested concentration of glyphosate. *C. curticei*, *O. circumcincta* and *H. contortus* showed similar statistical significance in both pesticides tested (there was high statistical significance (*p<*0.0001) at both concentrations of glyphosate and only at higher tested concentration of tolylfluanid). *C. curticei* and *H. contortus* larvae were found dead, spiral shaped and without movement at all concentrations tested, spiral shape was not observed in other two tested larvae. *O. circumcincta* larvae reacted to pesticides exposure very quickly; rapid death was recorded on second day of experiment at both concentrations of glyphosate and at higher tested concentration of tolylfluanid. From four tested small ruminant parasites (L3), *O. circumcincta* larvae seem to be the most sensitive ones and need further research.

## Introduction

Pesticides are frequently used in commercial agriculture to increase production despite their proven negative side effects on consumer health. The very significant impact of European legislation on the authorization of plant protection products (Directive 91/414/EEC) has resulted in withdrawal of 704 out of total 889 active substances assessed so far. There was observed at least one health side-effect including carcinogenicity, reproductive and neuro-developmental disorders, as well as endocrine disruption after acute or chronic exposure of 84 approved active substances (Karabelas *et al*., 2009).

All relevant living forms (birds and mammals, aquatic, bees and non-target arthropods, soil micro- and macro-organisms, non-target plants and sewage treatment) are included in the risk assessment of pesticides (it is ecotox section in Draft Assessment Report prepared by Notifier and reviewed by EFSA). Parasites and their development stages, frequently present in animal dung, get to the environment (e.g., surface layer of soil) by manuring and can be easily exposed to pesticides applied on crops. That is why the aim of this study was to observe the *in vitro* effect of two commercial pesticide products (containing glyphosate, resp. tolylfluanid) on larval stages of four common small ruminant parasite species. According to obtained results to assess whether the tested parasites are suitable for the risk assessment of pesticides and/or chemical substances.

The experiment included *Cooperia curticei*, *Ostertagia circumcincta*, *Haemonchus contortus* and *Trichostrongylus axei*. Product, containing glyphosate, is used as herbicide and second commercial product, containing tolylfluanid, was used as fungicides. The use of Plant Protection Products (PPP) containing active substance tolylfluanid is nowadays prohibited in Europe on the basis of commission restriction Nr. 2007/322/EC (Official Journal of the European Union).

## Materials and metods

### Chemicals

In our experiment we used following chemical substances: Herbicide Roundup Rapid® (Monsanto Europe S.A., Belgium) with the active substance glyphosate 450 g/L product and fungicide Euparen Multi® (Bayer AG, Germany) with the active substance tolylfluanid 500 g/L product.

There were two concentrations tested for each product (registered maximal concentration of active substance per ha and 10 times higher concentration) vs. control group (distillated water) in this study. In the case of glyphosate, we used concentrations of 1.4% and 14% and in the case of tolylfluanid it was 0.25% and 2.5% concentration.

### Parasite cultures

Larvae (L3) of *Cooperia curticei*, *Ostertagia circumcincta*, *Haemonchus contortus* and *Trichostrongylus axei* were obtained from Veterinary Laboratories Agency (Weybridge, UK). They were stored under the temperature of 4 °C until the use.

### Method of testing

Total amount of 500 larvae individuals were dosed with automatic micropipette per each tested concentration to Petri dishes (diameter of 40 mm). The number of larvae was microscopically calculated and if needed, (when number of individuals per a Petri dish was not reached) larvae were dosed again to reach correct value.

After application of pesticide concentrations (distilled water in control group), Petri dishes were incubated at average temperature of 27 °C in laboratory incubator (TER 80). Effects were regularly observed by using microscope (Nikon) every day for 42 days. If the effect of pesticides was present, larvae were found dead, without movement after microscope lighten.

### Data analysis

Results were statistically evaluated by using the Contingency table (GraphPad Prism® 3), where *p*<0.05 was regarded as significant.

## Results

From four tested ruminant parasites, *T. axei* larvae were the most resistant ones to tolylfluanid exposure – no statistical significance was observed in any tested concentration of tolylfluanid after 42 days of exposure (*p*=1.000). In the case of glyphosate, 100% of dead larvae were found on 33^rd^ day of experiment at higher concentration, resp. on 37^th^ day at lower tested concentration.

Survival of *C. curticei*, *O. circumcincta* and *H. contortus* showed similar statistical significance in both pesticides tested (experiment vs. control groups), where high statistical significance (*p<*0.0001) was observed in both concentrations of glyphosate and only at higher tested concentration of tolylfluanid.

Larvae of *O. circumcincta* reacted to pesticides exposure very fast; rapid loss of live larvae (*p<*0.001) was recorded on 2^nd^ day of experiment at both concentrations of glyphosate and at higher tested concentration of tolylfluanid.

Larvae of *C. curticei* were found dead (*p<*0.001) on 22^nd^ day of experiment at 1.4% glyphosate and on 19^th^ day at 14% glyphosate. Both concentrations of glyphosate were significantly toxic to *H. contortus* larvae on 5^th^ day of incubation (100% of larvae were found dead).

Tolylfluanid was significantly toxic to larvae of *C. curticei* and *H. contortus* (*p<*0.001) only at 2.5% concentration after 42 days of exposure.

**Figure 1:**
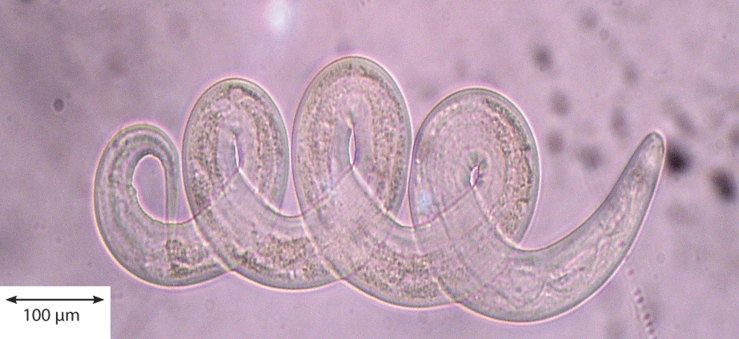
Spiral shape of the dead larvae of *Haemonchus contortus*.

Dead larvae of *C. curticei* and *H. contortus* were spiral shaped and without movement at both products tested (Figure 1), while spiral shape was not observed in other two larvae tested.

## Discusion

Chemical substances get to the environment by human activity in different ways. The most dangerous are those with long persistency and cumulative properties (Legáth *et al*., 1997). Parasites circulate in the environment among intermediate and definitive hosts (Ciberej *et al*., 2001), so it means they are exposed to environmental pollution (Hronec *et al*., 2002). Parasites may be exposed in external environmental conditions (e.g., egg stages on the field) or in the animal body (intake of contaminated food and water).

Parasites react to chemical exposure in different ways. There was observed low prevalence of small ruminant gastrointestinal nematodes (larval stages) on the pastures contaminated with heavy metals (mercury dominated) in Spiš region (Krupicer, 1995). Papajová (2001) observed significant *in vitro* ovocidal effect of several disinfectants approved for use in farms in Slovakia against *A. suum*. Legáth *et al*. (2005) found out that tolylfluanid was *in vitro* very effective against coccidial oocysts in concentration range 0.001–1.0 g/L after 28 days of exposure.

In our experiment, glyphosate was more toxic to parasite larvae than tolylfluanid. From four tested ruminant parasites (L3), larvae of *O. circumcincta* were the most sensitive and larvae of *T. axei* were the most resistant ones after 42 days of both pesticides exposure.

These results are new and there is a lack of information about the effect of pesticides on animal endoparasites. That is why there is a problem to discuss them with similar ones.

Investigation of the influence of several chemical substances (including glyphosate) on protozoan parasite *Perkinsus olseni* proliferation revealed that glyphosate inhibited *in vitro* proliferation of this parasite in a dose-dependent manner (Elandalloussi *et al*., 2005). Another experiment in which horsehair worms *Chordodes nobilii* (*Gordiida*, Nematomorpha) were exposed to glyphosate concentrations ranging between 0.1 and 8 mg/L for a short period of time detected that embryo development was not inhibited, but there was a significant decrease in the infective capacity of larvae derived from eggs that had been exposed to concentration ≥0.1 mg/L. Adult exposed to 1.76 mg/L of formulated glyphosate for 96 h shown a mortality of 50% (Achiorno *et al*., 2008).

Our results similarly to those of Legáth *et al*., Elandalloussi *et al*. and Achiorno *et al*. point out that the pesticides like glyphosate and tolylfluanid have some antiparasitic effect *in vitro* although they are primarily herbicides, resp. fungicides.

This study was focused on parasite larvae stage with assumption they are more sensitive to exposure than other life stages (Borošková and Dvorožňáková, 1997; Papajová, 2001). It is necessary to continue in survey of all metamorphosis phases of the most sensitive parasite species and their infective capacity. On the basis of complex research, the most sensitive parasite species with their stages could be set for risk assessment of different chemical compounds.

## References

[CIT0001] Achiorno CL, Villalobos C, Ferrari L (2008). Toxicity of the herbicide glyphosate to Chordodes nobilii (*Gordiida*, Nematomorpha). Chemosphere.

[CIT0002] Borošková Z, Dvorožňáková E (1997). The effect of cadmium on the immune behaviour of guinea pigs with experimental ascariasis. J Helminthol.

[CIT0003] Ciberej J (2001). Ekológia, in Starostlivosť o zver a choroby zveri (Ciberej J *ed*).

[CIT0004] Elandalloussi LM, Rodrigues PM, Afonso R, Leite RB, Nunes PA, Cancela ML (2005). Shikimate and folate pathways in the protozoan parasite, *Perkinsus olseni*. Mol Biochem Parasitol.

[CIT0005] Hronec O, Tóth J, Tomáš J (2002). Cudzorodé látky a ich riziká.

[CIT0006] Karabelas AJ, Plakas KV, Solomou ES, Drossou V, Sarigiannis DA (2009). Impact of European legislation on marketed pesticides – a view from the standpoint of health impact assessment studies. Environ Int.

[CIT0007] Legáth J, Bláha K, Čermáková T, Dolinay Š, Hufnagelová B, Kočišová A, Košuth P, Kotleba J, Legáth Ľ, Majláthová Ľ, Markovič J, Mlynarčíková H, Murín M, Ondrašovič M, Prosbová M, Škulínková K, Sokol J, Toporčák J (1997). Odhad miery rizika chemických látok pre domáce, hospodárske a voľne žijúce zvieratá, včely a vodné živočíchy.

[CIT0008] Legáth J, Vasil'ková Z, Krupicer I, Sabo R (2005). Účinok fungicídu tolylfluanidu a herbicídu bentazónu na atenuované oocysty kokcídií. Status Veterinarius.

[CIT0009] Krupicer I (1995). Vplyv imisií ťažkých kovov s dominanciou ortuti na priebeh pasienkových helmintóz oviec. Veterinární Medicína.

[CIT0010] Official Journal of the European Union [webpage on the internet] http://eur-lex.europa.eu/LexUriServ/LexUriServ.do?uri=OJ:L:2007:119:0049:0050:EN:PDF.

[CIT0011] Papajová I (2001). Pôsobenie vybraných abiotických a biotických faktorov prostredia na tenacitu vajíčok modelového nematóda Ascaris suum. Thesis (*Dissertation*).

